# Biochemical and Molecular Basis of Chemically Induced Defense Activation in Maize against Banded Leaf and Sheath Blight Disease

**DOI:** 10.3390/cimb46040192

**Published:** 2024-04-02

**Authors:** Shah Mahmood Hamidi, Shweta Meshram, Aundy Kumar, Archana Singh, Rajbir Yadav, Robin Gogoi

**Affiliations:** 1 Indian Council of Agricultural Research—Plant Pathology, Indian Agricultural Research Institute, New Delhi 110012, India; shahhamidi@yahoo.com (S.M.H.); kumar@iari.res.in (A.K.); 2 Department of Plant Pathology, School of Agriculture, Lovely Professional University, Phagwara 144402, Punjab, India; 3 Indian Council of Agricultural Research—Biochemistry, Indian Agricultural Research Institute, New Delhi 110012, India; sarchana19@gmail.com; 4 Indian Council of Agricultural Research—Genetics, Indian Agricultural Research Institute, New Delhi 110012, India; rajbiryadav@yahoo.com

**Keywords:** fungicides, *Rhizoctonia solani* f. sp. *sasakii*, defense inducers, antioxidant enzymes activity, defense gene expression

## Abstract

Maize is the third most vital global cereal, playing a key role in the world economy and plant genetics research. Despite its leadership in production, maize faces a severe threat from banded leaf and sheath blight, necessitating the urgent development of eco-friendly management strategies. This study aimed to understand the resistance mechanisms against banded leaf and sheath blight (BLSB) in maize hybrid “Vivek QPM-9”. Seven fungicides at recommended doses (1000 and 500 ppm) and two plant defense inducers, salicylic acid (SA) and jasmonic acid (JA) at concentrations of 50 and 100 ppm, were applied. Fungicides, notably Azoxystrobin and Trifloxystrobin + Tebuconazole, demonstrated superior efficacy against BLSB, while Pencycuron showed limited effectiveness. Field-sprayed Azoxystrobin exhibited the lowest BLSB infection, correlating with heightened antioxidant enzyme activity (SOD, CAT, POX, β-1,3-glucanase, PPO, PAL), similar to the Validamycin-treated plants. The expression of defense-related genes after seed priming with SA and JA was assessed via qRT-PCR. Lower SA concentrations down-regulated SOD, PPO, and APX genes but up-regulated CAT and β-1,3-glucanase genes. JA at lower doses up-regulated CAT and APX genes, while higher doses up-regulated PPO and β-1,3-glucanase genes; SOD gene expression was suppressed at both JA doses. This investigation elucidates the effectiveness of certain fungicides and plant defense inducers in mitigating BLSB in maize hybrids and sheds light on the intricate gene expression mechanisms governing defense responses against this pathogen.

## 1. Introduction

The importance of maize (*Zea mays* L.) as a vital food and industrial crop in India is evident, with an annual production of 27.82 million metric tons (MMTs) and an average national yield of 3.02 tons per hectare [1,2]. The production of maize is impacted by various biotic stresses, with susceptibility to diseases being a significant deterrent to achieving high grain yields [3]. Banded leaf and sheath blight (BLSB) stands out as a major disease that is particularly prevalent in tropical regions, notably in south and south-east Asian countries, including India. Under favorable conditions, the disease can result in losses of up to one hundred percent [1,4,5,6,7].

To manage BLSB, various strategies can be employed, and chemical approaches involving fungicides have proven to be comparatively effective. However, these methods are not eco-friendly, necessitating the exploration of alternative strategies. The resistance of the host plant plays a crucial role in disease management. Despite continuous efforts, developing resistance against BLSB remains a challenge, as no naturally resistant host plants have been identified. Limited genetic variation for resistance further complicates breeding programs [8]. Unfortunately, true resistance sources against BLSB are scarce, and genetic variability for resistance in maize is limited [8,9,10,11]. In the absence of BLSB-resistant maize varieties, chemically induced disease resistance is considered an alternative approach.

Salicylic acid (SA) functions as a vital plant growth regulator, playing a significant role in the signaling pathways triggered by diverse biotic and abiotic stresses [12]. It has been identified as an endogenous regulatory signal molecule, activating the general defense mechanisms of plants [13,14,15]. On the other hand, jasmonic acid (JA) and its derivatives are pivotal in plant responses to various stresses [16]. Systemic acquired resistance (SAR) is a highly desirable form of resistance that protects against a broad spectrum of related or unrelated pathogens. SAR involves the generation of multiple signals at the site of primary infection, which arms distal portions against subsequent secondary infections [17]. SA is not only essential for pathogen-induced SAR, as none of the identified chemical inducers can induce SAR in SA-deficient backgrounds.

In this context, exploring chemically induced resistance using compounds like salicylic acid (SA) and jasmonic acid (JA) emerges as a promising strategy [18,19]. Understanding the role of these inducers and their application methods is crucial in eliciting systemic acquired resistance (SAR) and activating defense mechanisms in plants [20]. However, the use of fungicides and chemical defense activators in maize, particularly their impact on biotic stress conditions, remains inadequately explored [21].

Reactive oxygen species (ROS) such as H_2_O_2_ are continuously generated in plant tissues as by-products of several metabolic processes [22]. To cope with ROS, plant cells possess an antioxidative system consisting of both enzymatic and non-enzymatic antioxidants [23]. SOD is considered to be the first line of defense against ROS [24]. CAT and APX are responsible for the scavenging of H_2_O_2_. CAT converts H_2_O_2_ to H_2_O and O_2_ and APX catalyzes the reduction of H_2_O_2_ using ascorbate as an electron donor. Other peroxidases, including guaiacol peroxidase (GPX), are also involved in H_2_O_2_ elimination [25]. Peroxidase (POX) oxidizes phenolics to more toxic quinones and generates hydrogen peroxide. The last step in the synthesis of lignin and suberin has been proposed to be catalyzed by peroxidases. PAL catalyzes the first step of the phenylpropanoid pathway, leading to the synthesis of a wide variety of secondary metabolites including flavonoids, coumarins, hydroxycinnamoyl esters, and lignin [26]. Due to the nature and defense-related functions of these metabolites, the activation of PAL against abiotic and biotic stresses has been considered a part of the defensive mechanisms of plants [27,28,29,30].

This investigation aimed to uncover effective BLSB management using plant defense inducers and understand the mechanisms behind chemically induced disease resistance in maize. We investigated the potential of SA and JA as inducers of SAR against BLSB, shedding light on their mechanisms of action and optimal application strategies [18,19,31]. This research also focused on elucidating plants’ defense mechanisms involving pre-existing physical and chemical barriers, along with inducible defense responses [31]. Enzymes such as superoxide dismutases (SODs) play a critical role in defense against oxidative stress. Different SOD isoforms respond differently to inhibitors, and their localization in various cell compartments underscores their importance in defense mechanisms [32,33,34]. The objective was to assess the impact of seven fungicides and two plant defense inducers, namely salicylic acid (SA) and jasmonic acid (JA), against *Rhizoctonia solani* f. sp. *sasakii*. This comparative analysis aims to provide insights into the potential level of pathogen control achievable in vitro before transitioning to field applications. This dual approach aimed to provide a comprehensive understanding of the treatment’s impact on the pathogen under different growth conditions.

## 2. Materials and Methods

### 2.1. Layout of Experiments

The in vivo research work was conducted at the main field and net house of ICAR-IARI, Pusa campus, using a susceptible maize variety: “Vivek QPM-9”. The variety was procured from ICAR-Vivekananda Parvatia Krishi Anusandhan Sansthan, Almora, Uttarakhand, India, and raised during the kharif seasons of 2020, and 2021. The plot size was 4.5 m^2^ and contained 3 rows (row length: 3 m, R to R 75 cm); 13 to 15 plants/row were maintained. The in vitro experiments were conducted in the laboratories using the poisoned food technique [35]. Three replications were maintained in each treatment of fungicides in both PDA and PDB culture media and were then incubated at 28 °C in a BOD incubator. Each treatment, comprising fungicides in both PDA and PDB culture media, was replicated three times and maintained at 28 °C in a BOD incubator for the culture of *R. solani* f. sp. *sasakii* (Source Indian Type Culture Collection, ITCC Accession No. 6881), Division of Plant Pathology, IARI, New Delhi.

### 2.2. Isolation of the Pathogen and Mass Multiplication of Inoculum

The original culture for the research was obtained from ITCC (Accession No. 6881). The long-term stored culture was further cultured in potato dextrose agar (PDA), and the actively growing culture (mycelia) was used in the inoculation of maize seedlings, to pass through the host providing better revival with aggressiveness. The fungus was re-isolated on PDA and mass cultured in barley grains, which were used in the inoculation of the maize crops following a standard method [36]. The pure cultures were maintained in PDA slants at 4 ± 1 °C for use in this study. The mass culture of the pathogen was prepared using the method described by Ahuja and Payak [37]. Barley grains were soaked overnight in tap water, and the water was then drained out. Forty grams of water-soaked seeds were placed in 250 mL Erlenmeyer flasks, plugged, and autoclaved two times at 121.6 °C (15 lb) for 20 min. The sterilized barley grains were inoculated with a small quantity of 5–8-days-old fungal culture. The flasks were incubated at 27 ± 1 °C for 10–15 days and shaken at every 2–3 day interval to ensure uniform fungal growth in the grains (Table 1).

### 2.3. In Vitro Evaluation of Fungicides against Rhizoctonia solani f. sp. sasakii

The fungicides that were mentioned in 2.4 were evaluated in vitro at two different concentrations, 500 and 1000 ppm. The required quantity of the fungicides was put into the molten PDA medium and mixed thoroughly. The medium was poured into each Petri dish (90 mm diameter). After solidification, 5 mm discs of the 4–5-days-old actively grown fungus (mycelium) were cut out using a sterilized cork borer. The mycelium disc was placed at the center of Petri plates, sealed with parafilm and incubated at 28 ± 1 °C. The radial growth of the fungus was recorded treatment wise when complete growth was attained in the control (untreated) plates. In the case of liquid media (PDB), the desired amount of the fungicides was mixed with 40 mL PDB in 100 mL conical flaks. Then, a 5 mm disc of the fungus mycelium was put in the poisoned PDB. The flasks were incubated at 28 ± 1 °C for 14 days in a shaker incubator (ISF-1-W, Kuhner, Birsfelden, Switzerland) maintained at 150 rpm. The mycelial ball was harvested by filtering through Whatman filter paper. Then, the mycelial ball was placed on the glass Petri plates covered with a tissue paper and kept there for 4–6 h to air dry. The ball was further dried overnight in an electric oven at 50 °C. The properly dried mycelia were weighed using an electronic balance (Sartorius, Göttingen, Germany). Percent (%) inhibition was calculated for each treatment [38] using the formula I = (C − T)/C × 100, where I = Percent inhibition (%), C = Colony diameter in control (mm), and T = Colony diameter in treatment (mm).

### 2.4. Plant Inoculation with the Pathogen and Spray of Fungicides

**Figure 1:**
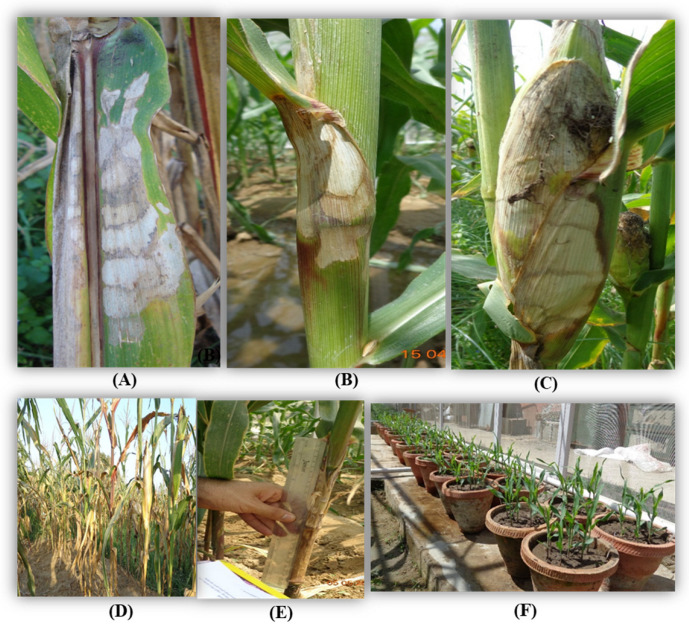
Symptoms of banded leaf and sheath blight (BLSB) on (**A**) leaf, (**B**) sheath, and (**C**) cob. (**D**) Artificially inoculated disease field. (**E**) Measurement of lesion length. (**F**) Treated seeds sown in pots and kept under net house conditions.

Inoculation was performed on the 35-days-old maize plants [39] with a barley grain culture of *R. solani.* f. sp. *sasakii*. Three barley grains bearing fungal mycelia were carefully inserted by using fingers between the stalk and sheath at the second or third internode from the soil surface [37]. The fungicides, namely Hexaconazole at 0.1%, Carbendazim at 0.1%, Validamycin at 0.1%, Tebuconazole at 0.05%, Trifloxystrobin 25% + Tebuconazole 50% at 0.05%, Azoxystrobin at 0.05%, and Pencycuron at 0.1%, were sprayed on individual plants 3 days after inoculation (DAI) using hand sprayers (Super Garden, Coimbatore, India). All precautions were taken at the time of spraying to avoid drifting to the adjacent treatments (Figure 1). Leaf and sheath samples showing typical symptoms of BLSB, such as dark lesions with characteristic banding on the leaves and sheath necrosis, were collected for further study (Figure 1).

### 2.5. Evaluation of Fungicides against BLSB Disease and Grain Yield

The efficacy of the fungicides on BLSB disease of maize was determined. The inoculated and fungicide-sprayed plants were monitored for the appearance of symptoms and disease progression regularly on a weekly basis. The percent (%) disease severity, virulence index, and length of infected area in each plant were recorded on 30 DAI. Records of the disease intensity were calculated using a 1–5 disease rating scale (Table 2) [40]. As per the new rating scale, the percent disease index (PDI) was calculated using the formula given by McKinney [41].

According to the PDI disease rating plants could be classified as resistant (R), moderately resistant (MR), moderately susceptible (MS), and susceptible (S). For grain yield (q/ha), the total weight (kg) of the fully matured harvested cobs was recorded and plotted by treatment. The shelling of the grains was initially performed and the moisture content of the grains was recorded by using a moisture meter (AgraTronix MT-PRO, Agratronix Corporate, Streetsboro, OH, USA). Grain yield was calculated by applying three formulas [42].

### 2.6. Assay of Enzyme Activity in Maize Plants Treated with Fungicides

The enzyme activity in the fungicide-treated maize plants was evaluated. For the healthy group, young leaves of 30–35-days-old plants were collected at specific intervals: 3 days before fungicide spray, i.e., at the time of pathogen inoculation; on the day of fungicide application (day 0); and subsequently 5 days later. In the early morning, upper leaves were cut using sterilized scissors, promptly placed in the pre-labeled ice bags, and stored at −80 °C.

Subsequently, superoxide dismutase (SOD) maize leaf tissue (1 g) was ground in 5 mL grinding media (0.1 M phosphate buffer, pH 7.5, with 0.5 mM EDTA). After centrifugation, the supernatant was used for the SOD assay. The reaction mixture contained phosphate buffer, L-methionine, NBT, riboflavin, and enzyme extract. Absorbance was measured at 560 nm and a standard curve was prepared using known concentrations of a substance relevant to the SOD assay [43]. Catalase (CAT) activity was measured by following Aebi’s method (1984) with modifications. Protein estimation was performed using the Bradford method. CAT activity was assayed in a 3 mL reaction mixture (50 mM potassium phosphate buffer, 12.5 mM hydrogen peroxide, 100 µL enzyme extract), and the decomposed H_2_O_2_ was quantified at 240 nm; activity was calculated within a specific range of 5–12 U/mg and was prepared using known concentrations of bovine serum albumin (BSA) [44,45,46]. A peroxidase (POX) leaf sample (1 g) was ground in 5 mL grinding media. The supernatant obtained after centrifugation was used for the POX assay; activity was determined at 470 nm using a reaction mixture containing phosphate buffer, guaiacol, enzyme extract, and H_2_O_2_, and then a standard curve was prepared using known concentrations of guaiacol [47]. Phenylalanine ammonia-lyase (PAL) activity was assayed with a reaction mixture containing L-phenylalanine, borate buffer, and enzyme extract; then absorbance was measured at 290 nm and a standard curve was prepared using known concentrations of trans-cinnamic acid, which is the product of the PAL reaction [48,49]. Polyphenol oxidase (PPO) activity was estimated by recording absorbance at 495 nm in a reaction mixture containing phosphate buffer, proline, and catechol and a standard curve using known concentrations of catechol [50]. β-1,3-glucanase activity was determined by incubating enzyme extract with laminarin solution and measuring the absorbance at 500 nm after adding dinitro salicylic reagent and using a standard curve of D-glucose [51] (Supplementary Figures S1–S3 Annexure I).

### 2.7. Expression Study of Salicylic Acid (SA) and Jasmonic Acid (JA) Genes of Maize during Infection

Maize seeds (variety Vivek QPM 9) were treated with 50 and 100 ppm concentrations of SA and JA and sown in the 10 cm diameter pots under net house condition Figure 1 shows that the average natural temperature during kharif season ranges from 22 to 25 °C. Two seedlings were maintained in each pot. For gene expression analysis, ten-day-old seedlings were collected. Specific primers for each gene were designed by using BioEdit 7.2.5 and IDT Primer Quest software (eu.idtdna.com/Primerquest/Home/Index, accessed on 20 May 2018), with reference sequences for the maize genes obtained from NCBI (Table 3).

### 2.8. Primer Validation, RNA Isolation, cDNA Synthesis, and qRT-PCR Analysis of Maize Genes

The designed primers of SOD (ZM_SOD), β-1,3-glucanase (ZM_Glucan), PPO (ZM_PPO), ascorbate peroxidase (ZM_APX), CAT (ZM_CAT), and PAL were validated following Williams’s [52] protocol with minor adjustments in annealing temperature. RNA was isolated from the SA- and JA-treated and control maize seedlings. The Pure LinkTM RNA Mini Kit was used for extraction, purification, and quantification via a NanoDrop spectrophotometer. First-strand cDNA synthesis was performed by using the ImProm-II™ Reverse Transcription System kit. SYBR green-based qRT-PCR analysis was carried out by employing the primers listed in Table 2. Gene expression was confirmed via agarose gel electrophoresis (Supplementary Figure S4). The amplification conditions consisted of an initial denaturation step at 95 °C for 10 min, followed by 40 cycles of denaturation at 95 °C for 15 s, annealing at 60 °C for 30 s, and extension at 72 °C for 30 s. For each gene, qRT-PCR reactions were performed in two technical replicates across two independent biological replicates. Relative gene expression was quantified using the comparative Ct method (2^−ΔΔCt^) given by Livak [52]. Melt curve has been provided in supplemental file Supplementary Table S1.

### 2.9. Statistical Analysis

All the field and net house experiments were conducted in a randomized block design (RBD). The laboratory experiments were performed in a completely randomized design (CRD). Statistical analysis was conducted using analysis of variance (ANOVA) and the post hoc Tukey honestly significant difference (HSD) test, methods suitable for identifying pairwise differences when multiple groups are involved. We used online software (https://astatsa.com/OneWay_Anova_with_TukeyHSD/ accessed on 1 December 2023).

## 3. Results

The present study aimed to provide insights into managing BLSB disease by employing plant defense inducers and comprehending the biochemical mechanisms behind disease resistance in maize. Considering the non-availability of BLSB-resistant maize varieties, chemically induced disease resistance was explored as an alternative. Initially, seven fungicides were assessed in vitro against *R. solani* f. sp. *sasakii*. Furthermore, both fungicides and plant defense inducers were evaluated in vivo to determine their efficacy in restricting BLSB disease in maize.

### 3.1. In Vitro Evaluation of Fungicides against Rhizoctonia solani f. sp. sasakii

**Figure 2:**
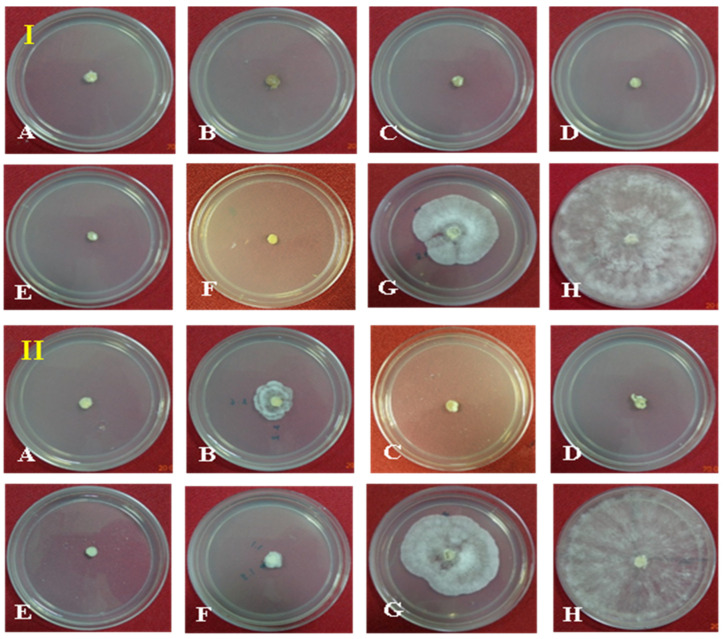
Effect of fungicides (1000 ppm, (**I**) and 500 ppm, (**II**)) on redial growth of *Rhizoctonia solani* f. sp. *sasakii* in vitro: (**A**) Hexaconazole, (**B**) Carbendazim, (**C**) Validamycin, (**D**) Tebuconazole, (**E**) Trifloxystrobin + Tebuconazole (Nativo), (**F**) Azoxystrobin, (**G**) Pencycuron, and (**H**) control (untreated).

Seven different fungicides at two dosages (500 and 1000 ppm) were evaluated against the pathogen in PDA as a positive control. At 1000 ppm, 100% growth inhibition was observed with treatment using the fungicides Hexaconazole, Carbendazim, Validamycin, Tebuconazole, Trifloxystrobin + Tebuconazole (Nativo), and Azoxystrobin as compared to the control. The lowest growth inhibition was observed in the treatment with Pencycuron (52.04%). At 500 ppm, the highest growth inhibition of the pathogen was observed in the treatments with Hexaconazole, Validamycin, Tebuconazole, and Trifloxystrobin + Tebuconazole (Nativo) (100%), followed by Azoxystrobin (92.19%) and Carbendazim (84.63%). The lowest growth inhibition was observed in the treatment with Pencycuron (48.52%) (Table 3, Figure 2).

**Figure 3:**
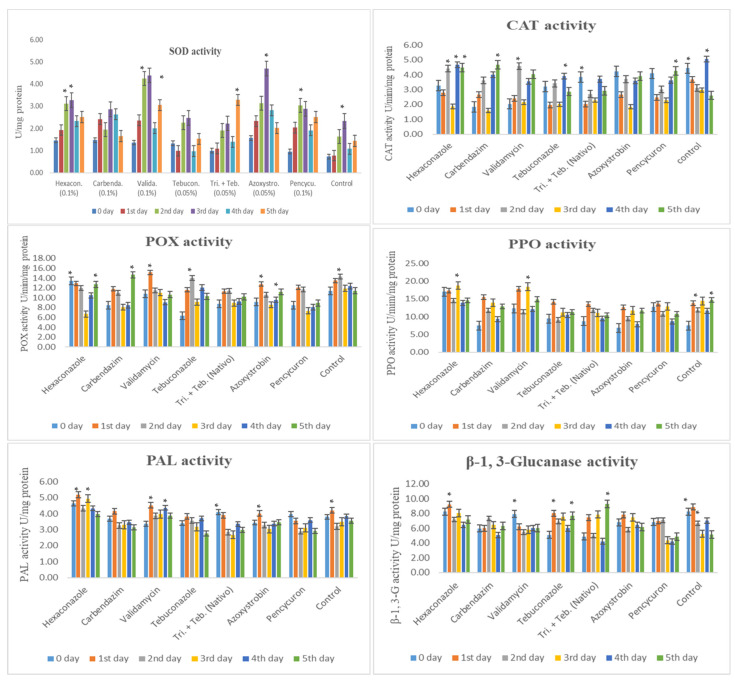
Superoxide dismutase (SOD), catalase (CAT), peroxidase (POX), polyphenol oxidase (PPO), phenylalanine ammonia-lyase (PAL), and β-1,3-glucnase activity in maize (Vivek QPM-9) inoculated with *R. solani* f. sp. *sasakii* after fungicide application. Activity recorded at different inoculation days in an of interval 0 to 5th day after inoculation. The post hoc Tukey’s HSD test (*p* < 0.01) indicates a significant difference (*) in enzyme activity.

The percent reduction in the mycelial mass of the pathogen was also observed to be different in different fungicidal treatments as compared to the untreated control in PDB. At 1000 ppm concentration maximum reduction in mycelial mass was observed in the cases of Hexaconazole, Carbendazim, Validamycin, Tebuconazole, Trifloxystrobin + Tebuconazole (Nativo), and Azoxystrobin (100%), whereas the lowest reduction was observed in the Pencycuron treatment (95.30%). At 500 ppm, the maximum reduction in mycelial mass was observed in the treatments with Hexaconazole, Carbendazim, Validamycin, Tebuconazole, Trifloxystrobin + Tebuconazole (Nativo), and Azoxystrobin (100%), whereas the lowest reduction was observed in the Pencycuron treatment (94.71%) (Table 4, Figure 3).

### 3.2. Effect of Fungicides on BLSB Disease of Maize and Grain Yield

The maximum lesion length was observed on the plants sprayed with Hexaconazole (0.1%) (45.76 cm), followed by the plants sprayed with Pencycuron (0.1%) (44.83 cm), Trifloxystrobin + Tebuconazole (Nativo, 0.05%) (37.20 cm), and Carbendazim (0.1%) (36.72 cm). The lowest length of disease lesions was observed in the plants sprayed with Azoxystrobin (0.05%) (31.24 cm), Tebuconazole (0.05%) (32.22 cm), and Validamycin (0.1%) (33.94 cm). The lesion length in untreated (water sprayed) plants was 55.38 cm.

The maximum disease score on the 1–5 scale was observed in the plants sprayed with Pencycuron (0.1%) (3.91), followed by plants sprayed with Carbendazim (0.1%) (3.49), Hexaconazole (0.1%) (3.41), Validamycin (0.1%) (3.23), and Tebuconazole (0.05%) (3.14), whereas the lowest disease scores were observed in plants sprayed with Azoxystrobin (0.05%) (2.81) and plants sprayed with Trifloxystrobin + Tebuconazole (Nativo, 0.05%) (2.97).

The highest percent disease index (PDI) was recorded in the plants sprayed with Pencycuron (0.1%) (77.73%), followed by the plants sprayed with Carbendazim (0.1%) (69.72%), Hexaconazole (0.1%) (68.27%), Validamycin (0.1%) (64.55), and Tebuconazole (0.05%) (62.79%), whereas the lowest percent disease index was recorded in the plants treated with Azoxystrobin (0.05%) (56.2%) and the plants treated with Trifloxystrobin + Tebuconazole (Nativo, 0.05%) (59.36%). The percent disease index for the untreated control plants was recorded as 95.10%.

The highest grain yield was obtained in the plants treated with Azoxystrobin (0.05%) (58.35 q/ha). followed by the plants treated with Tebuconazole (0.05%) (56.61 q/ha), Validamycin (0.1%) (53.93 q/ha), and Carbendazim (0.1%) (50.16 q/ha), whereas the lowest grain yield was recorded in the plants treated with Pencycuron (0.1%) (47.12 q/ha), Trifloxystrobin + Tebuconazole (Nativo, 0.05%) (48.66 q/ha), and the plants treated with Hexaconazole (0.1%) (49.44 q/ha). In the untreated control plants, the grain yield was recorded as 44.93 q/ha (Table 5).

### 3.3. Estimation of Biochemical Defense-Related Enzymes in Maize Treated with Fungicides

This study examined the impact of fungicides on maize’s biochemical defense enzymes against *R. solani* f. sp. *sasakii* (Figure 3).

Superoxide dismutase (SOD) activity was the highest with Azoxystrobin (0.05%) at 3 DAI, followed by Validamycin (0.1%), Hexaconazole (0.1%), and Pencycuron (0.1%) at 2 and 3 DAI. Trifloxystrobin + Tebuconazole showed induction at 5 DAI. Tebuconazole (0.05%) and Carbendazim (0.1%) had lower SOD elevations. Catalase (CAT) activity peaked in the control group at 0 and 4 DAI. Azoxystrobin (0.05%), Validamycin (0.1%), Tebuconazole (0.05%), Carbendazim (0.1%), and Pencycuron (0.1%) showed either equal or lower activity, especially at 1 and 4 DAI.

Peroxidase (POX) activity was the highest with Validamycin (0.1%) and Carbendazim (0.1%) at 1 and 5 DAI, while Hexaconazole (0.1%) exhibited the least activity on the 3rd day. Polyphenol oxidase (PPO) activity peaked with Validamycin (0.1%) and Hexaconazole (0.1%) at 1 and 3 DAI. Azoxystrobin (0.05%), Tebuconazole (0.05%), and Pencycuron (0.1%) showed lower activity levels.

Phenylalanine ammonia-lyase (PAL) activity was notably higher with Hexaconazole (0.1%) and Validamycin (0.1%) on the 1st and 3rd days. The other treatments showed similar patterns to the control. β-1,3-glucanase (β-1,3-G) activity was notably high with Trifloxystrobin + Tebuconazole (Nativo, 0.05%) at 5 DAI, and Tebuconazole (0.05%) at 1 and 5 DAI. Conversely, the Pencycuron (0.1%), Azoxystrobin (0.05%), Validamycin (0.1%), and Tebuconazole (0.05%) treatments all showed lower activity compared to the control.

### 3.4. Effects of Salicylic Acid and Jasmonic Acid Seed Priming on Expression of Defense-Related Genes in Maize

**Figure 4:**
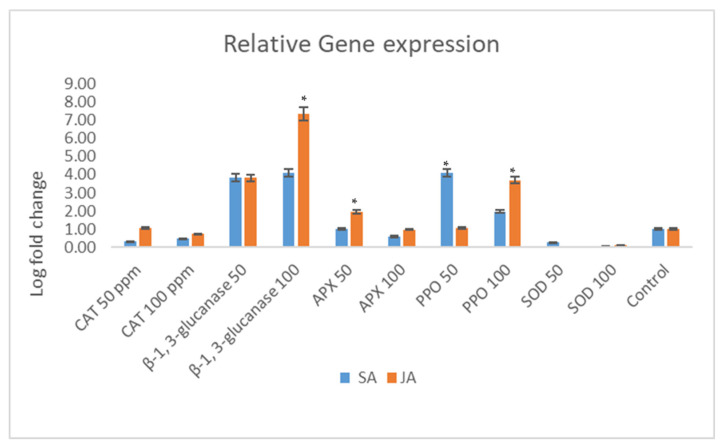
Relative expression of selected defense genes in maize treated with salicylic acid (SA) and jasmonic acid (JA). Enzymes include superoxide dismutase (SOD), polyphenol oxidase (PPO), ascorbate peroxidase (APX), catalase (CAT), and β-1,3-glucanase (β-1,3-gluc). The post hoc Tukey’s HSD test (*p* < 0.01) indicates a significant difference (*) in gene expression between the samples treated with salicylic acid and jasmonic acid.

This study investigated the influence of seed treatments with salicylic acid (SA) and jasmonic acid (JA) on the relative expression of defense-related genes in maize (Figure 4).

The relative expression of the maize superoxide dismutase (SOD) gene was modulated by the SA and JA treatments. Elevated SA concentrations led to a reduction in SOD expression, indicating a suppressive effect of SA on maize SOD expression. In contrast, escalating doses of JA resulted in a heightened expression of the SOD gene. The expression of the maize polyphenol oxidase (PPO) gene responded differently to the SA treatment, with increased concentrations of SA further diminishing gene expression. Conversely, the JA treatment elicited a robust expression of the PPO gene, suggesting its potential involvement in maize defense mechanisms. Particularly, at a concentration of 100 ppm, JA induced a substantial four-fold increase in PPO gene expression compared to the 50 ppm JA treatment.

In contrast, the relative expression of the maize ascorbate peroxidase (APX) gene exhibited divergent responses to SA and JA. Higher SA concentrations (100 ppm) halved APX expression relative to the control, while the JA treatment at 50 ppm nearly doubled APX expression. Intriguingly, APX expression remained stable at JA concentrations of 100 ppm. The expression of the maize catalase (CAT) gene showed a dose-dependent response to SA and JA treatments. CAT gene expression increased by approximately 3-fold and 3.2-fold with the SA and JA treatments at 50 ppm, respectively. However, as the SA/JA concentrations increased, there was a proportional decrease in CAT gene expression, indicating a dose–response relationship.

Distinct expression patterns were observed for the maize β-1,3-glucanase (β-1,3-gluc) gene in response to the SA and JA treatments. At 50 ppm, β-1,3-glucanase expression was markedly elevated. While escalating SA concentrations suppressed β-1,3-glucanase expression, the JA treatment led to its upregulation. Notably, the JA treatment at 100 ppm resulted in a remarkable 27-fold increase in gene expression, highlighting the involvement of the JA pathway in enhancing defense against maize pathogens (Figure 4).

Overall, the assay results reveal differential enzyme induction patterns across treatments, with Azoxystrobin exhibiting a superior induction of SOD activity, Validamycin and Carbendazim showing the highest POX activities, and Hexaconazole and Validamycin demonstrating elevated PAL activities compared to the other treatments.

## 4. Discussion

The current study aimed to investigate the management of BLSB disease in maize using plant defense inducers while exploring the biochemical and molecular mechanisms of disease resistance. Previous research has demonstrated the efficacy of Carbendazim against *Rhizoctonia solani*, the causal agent of BLSB, both in vitro and in vivo [53]. Similar outcomes have been reported, with reduced BLSB severity following foliar spray of Carbendazim on maize [54]. Another study reported the minimal effect of Strobilurins (Azoxystrobin) on *R. solani* mycelial growth in vitro [55]. Our findings indicate that, among the fungicides tested, Strobilurins and Carbendazim were more effective, aligning with previous research documenting excellent disease control with Strobilurins, Triazoles, and Benzimidazoles [56].

In addition to gene expression analysis, we evaluated the activity of key defense enzymes. These findings suggest that different fungicides may elicit distinct defense responses in maize, emphasizing the importance of selecting appropriate fungicides for effective disease management. A similar enzymatic induction was also recently reported in maize against another foliar pathogen, *Colletotrichum graminicola*, which resulted in local and systemic resistance, emphasizing the enzyme-mediated defense mechanisms in maize [57].

Superoxide dismutase (SOD) activity has delivered effective mitigations of reactive oxygen species during pathogenic infection in several studies [23,24]. Azoxystrobin, a quinone outside inhibitor (QoI), has been demonstrated to posses a capacity to activate reactive oxygen species, scavenge H_2_O_2_, and enhance the synthesis of secondary metabolites, as is reflected in the heightened activities of defense-related enzymes like SOD, catalase (CAT), and β-1,3-glucanase, akin to the antifungal antibiotic Validamycin. Conversely, salicylic Acid (SA) application on maize seeds has negatively impacted the expression of SOD and polyphenol oxidase (PPO) genes in a concentration-dependent manner, suggesting a potential damage-induced accumulation of H_2_O_2_ [22]. Some studies have also highlighted that SOD has minimal effects on JA induction [58]. The constitutive expressions of pathogenesis-related proteins and antioxidant enzyme activities, including superoxide dismutase (SOD), peroxidase (POD), and β-1,3-glucanase, play a pivotal role in triggering maize resistance against various pathogens [59]. These proteins and enzymes are integral components of the plant’s defense machinery, contributing to the mitigation of oxidative stress and the degradation of fungal cell walls [22,59,60].

The nuanced responses of APX and CAT genes to different SA concentrations suggest a finely tuned balance in maize antioxidant defenses, with higher SA doses potentially inducing oxidative stress mitigation. Similar findings have been observed by previous studies in various forms of oxidative stress caused by abiotic factors in plants [25]. Furthermore, the dose-dependent decrease in CAT gene expression with rising SA levels implies a dynamic regulation of hydrogen peroxide scavenging mechanisms, which supports the results of previous studies [61] performed during plant stress and development. Meanwhile, the notable increase in β-1,3-glucanase gene expression at 50 ppm SA underscores the complex interplay between SA signaling and defense response activation in maize. These findings aligns with a recent study conducted in maize, where the phyllosphere microbiome modulated the physiology of plants through enzymatic interplay, which ultimately showed defense against the foliar pathogen *Exserohilum turcicum* [62].

Jasmonic acid (JA) application has displayed similar complexities, impacting the expression of genes associated with defense mechanisms [60]. JA negatively affected the expression of the SOD gene in maize, with increasing concentrations leading to an enhanced expression. This dynamic modulation indicates the sophisticated and adaptable nature of plant defense mechanisms under JA influence [58,63]. Additionally, JA application increased the expression of the β-1,3-glucanase gene, emphasizing its role in modulating defense against maize pathogens. The JA 100 ppm dose recorded a 27-fold increase in gene expression, suggesting the involvement of the JA pathway in enhancing defense against maize pathogens. This response is similar to that seen with other maize pathogens such as *maize Fusarium verticillioides* [64] and *Colletotrichum graminicola* [65].

**Figure 5:**
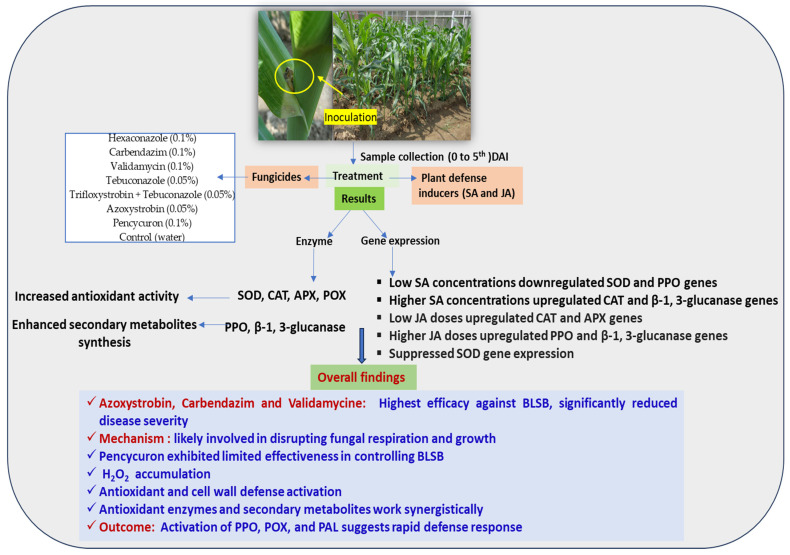
Summary of research on maize defense mechanisms against banded leaf and sheath blight. Investigation of the impact of fungicides and plant defense inducers on biochemical and molecular responses, providing insights into disease resistance in maize in present study.

This study reveals the multifaceted interactions between plant defense inducers and the intricate regulation of defense-related genes, providing valuable insights into the complex dynamics of plant responses to varying concentrations of these inducers. The findings contribute to our understanding of the biochemical and molecular mechanisms that govern disease resistance in maize (Figure 5). Our findings offer practical guidance for enhancing maize disease management, specifically against BLSB, by optimizing fungicide selection and application methods. The theoretical implications include advancing our understanding of plant defense mechanisms and plant–fungicide interactions, contributing to the broader agricultural science. However, the limitations include the focus on specific fungicides and enzymes, potentially limiting the generalizability of our findings to broader disease management contexts in maize. Therefore, future research could investigate the synergistic effects of fungicide combinations, integrate biological control methods, and assess the long-term impacts on soil health for sustainable maize disease management.

## 5. Conclusions

In conclusion, this research provides valuable insights into the management of banded leaf and sheath blight (BLSB) disease in maize. This study explores the biochemical elicitors that induce defense, offering potential avenues for disease control. Both salicylic acid (SA) and jasmonic acid (JA) have been demonstrated to show efficacy in suppressing the necrotrophic soil-borne phytopathogen *R. solani*. While seed priming with these inducers contributed to healthy seed germination, they did not significantly enhance the overall growth, development, and defense induction in maize plants. The artificial application of SA and JA was as effective and able to induce defense responses as chemical application. Hence, we suggest them as a better alternative to chemicals. Considering the resource-intensive nature of maize cultivation, this study sheds light on the prospect of reducing fungicide usage through a plant defense inducer-mediated host resistance approach in disease management. This not only holds promise for effective disease control but also aligns with the goal of promoting a safer and environmentally conscious agricultural environment.

## Figures and Tables

**Table 1:** List of fungicides used in this study along with their trade name.

S. No.	Common Name and (a.i. Formulation)	Trade Name	Chemical Name	Empirical Formula	Source
1	Hexaconazole 5% SC	Contaf Plus	2-(2,4-Dichlorophenyl)-1-(1H-1,2,4-triazol-1-yl) hexan-2-ol	C_14_H_17_C_l2_N_3_O	TATA Rallis India
2	Carbendazim 50% (*w*/*w*) WP	Bavistin	Methyl 1-2 benzimidazole carbamate	C_9_H_9_N_3_O_2_	BASF India
3	Validamycin 3% (*w*/*w*) L	Sheathmar-3	2,3-Dihydroxy-6 (hydroxymethyl)-4-[-4,5,6-trihydroxy 3 (hydroxymethyl) cyclohex-2-en-1 yl] amino cyclohexyl β-D glucopyranoside.	C_20_H_35_NO_13_	Dhanuka Agritech
4	Tebuconazole 25.9% (m/m) EC	Folicur	1-(4-Chlorophenyl)-4,4-dimethyl-3-(1H, 1,2,4-triazol-1-ylmethyl) pentan-3-ol	C_16_H_22_ClN_3_O	Bayer Crop Science
5	Trifloxystrobin 25% + Tebuconazole 50% (m/m) WG	Nativo	Benzeneacetic acid, (E,E)-alpha-(methoxylmino)-2-((((1-(3-trifluoromethyl) phenyl) ethylidene) amino) oxy) methy)-,methyl ester methyl (E)-2-[2-[6-	C_20_H_19_F_3_N_2_O_4_ and C_16_H_22_ClN_3_O	Bayer Crop Science
6	Azoxystrobin 23% (*w*/*w*) SC	Amistar	(2-Cyanophenoxy) pyrimidin-4-yl] oxyphenyl]-3-methoxyprop-2-enoate	C_22_H_17_N_3_O_5_	Syngenta India
7	Pencycuron 22.9% (*w*/*w*)	Monceren	1-(4-chlorbenzyl)-1-cyclopentyl-3-phenylurea	C_19_H_21_ClN_2_O	Bayer Crop Science

**Table 2:** Area under the disease progress curve (AUDPC) values for different disease severity categories at 30 days after inoculation.

PDI	AUDPC	Disease Severity (According to 1–5 Scale)
20.0	1050	Resistant (R) (Score: ≤2.0) (1 to 1.5) (PDI ≤ 40.0)
30.0		
50.0	1575	Moderately resistant (MR) (Score: 2.1–3.0) (PDI 40.1–60.0)
60.0		
70.0	2175	Moderately susceptible (MS) (Score:3.1–4.0) (PDI 60.1–80.0)
80.0		
90.0	2775	Susceptible (S) (Score: ≥4.0) (PDI ≥ 80.0)
100.0		

Note: The initial assessment was conducted at 0 days post-inoculation, followed by a subsequent assessment at 30 days. The average PDI for each interval was then multiplied by the corresponding time duration. The resulting values were summed to compute the AUDPC, employing the trapezoidal rule. ANOVA at 5% level of significance and post hoc Tukey’s HSD test statistics reveal that the treatment groups differ significantly. This comparative analysis offers insights into susceptibility and resistance levels across the treatments (F Statistic = 10.1453, *p*-value 0.0243).

**Table 3:** PCR primers used in this study.

Gene	Primer Details (5′-3′)	Size (bp)	Accession No
Superoxide dismutase	ZM_SOD (F *) 5′-AGT CAC CCA CCC CAT CCA AG-3′	146	NC_050102.1
	ZM_SOD (R ^#^) 5′-GTG CGG AGG AAT AGG GAG C-3′		
β-1,3 glucanase	ZM_Glucan (F) 5′-ATG GCG AGG CAG GGT GTC-3′	188	NC_050098.1
	ZM_Glucan (R) 5′-ACG CCG ATG GAT TGG ACT C-3′		
Polyphenol oxidase	ZM_PPO (F) 5′-CGT CCA AGA AGA CCA CCG T-3′	146	NC_050105.1
	ZM_PPO (R) 5′-ACT GGA CAG GCC GTT GAG CA-3′		
Ascorbate peroxidase	ZM_APX (F) 5′-ACC ATG AAG ACC CCC GTC GA-3′	118	
	ZM_APX (R) 5′-GGT AGA AGT CAG CGT AGG ATA G-3′		NC_050100.1
Catalase	ZM_CAT (F) 5′-ACG TGC GCC GAC TTC CTG-3′	180	
	ZM_CAT (R) 5′-GAA GAA GAC GGG GAA GTT GTT-3′		NC_050099.1
Phenylalanine ammonialyase	ZM_PAL (F) 5′-TCG AAC TGC AAC CGA AAG A-3′	108	NC_050096.1
	ZM_PAL (R) 5′-CAG CCA GGA TTG CCA GAA TA-3′		

* Forward, ^#^ Revers.

**Table 4:** Effect of fungicides on radial growth and mycelial mass of *Rhizoctonia solani* f. sp. *sasakii* inn PDA and PDB.

Treatments	Fungicides	Potato Dextrose Agar (PDA)				Potato Dextrose Broth (PDB)			
		Radial Growth * (mm) at	Inhibition (%) at	Radial Growth * (mm) at	Inhibition (%) at (500 ppm)	Mycelial Weight * (mg) at	Reduction (%) at (1000 ppm)	Mycelial Weight * (mg) at	Reduction (%) at (500 ppm)
		(1000 ppm)	(1000 ppm)	(500 ppm)		(1000 ppm)		(500 ppm)	
T1	Hexaconazole	0	100	0	100.00 (90.00)	0	100.00 (90.00) ^#^	0	100.00 (90.00)
			(90.00) ^#^						
T2	Carbendazim	0	100	13.5	84.63 (66.94)	0	100.00 (90.00)	0	100.00 (90.00)
			−90						
T3	Validamycin	0	100	0	100.00 (90.00)	0	100.00 (90.00)	0	100.00 (90.00)
			−90						
T4	Tebuconazole	0	100	0	100.00 (90.00)	0	100.00 (90.00)	0	100.00 (90.00)
			−90						
T5	Tri. + Teb. (Nativo)	0	100	0	100.00 (90.00)	0	100.00 (90.00)	0	100.00 (90.00)
			−90						
T6	Azoxystrobin	0	100	13.83	92.19 (76.67)	0	100.00 (90.00)	0	100.00 (90.00)
			−90						
T7	Pencycuron	43.17	52.04	46.33	48.52 (44.12)	15.17	95.3	23.7	94.71
			−46.2				−77.46		−76.78
T8	Control (untreated)	90	0	90	0	324.27	0	461.87	0
			0		0		0		0
C. D. (5%)		--	5.05	--	7.61	--	0.32	--	1.3
C. V.		--	3.95	--	6.37	--	0.24	--	0.96

* Data of the table are the means of three replications. ^#^ Data within parentheses are angular transformed values.

**Table 5:** Efficacy of fungicides on BLSB disease and grain yield of maize (Vivek QPM-9) under field conditions.

Tr.	Fungicide	Lesion Length * (cm)	Disease Score * (1–5 Scale)	PDI * (%)	Yield ^π^ (Q/ha)
T1	Hexaconazole (0.1%)	45.76	3.41	68.27 (56.44) ^#^	49.44
T2	Carbendazim (0.1%)	36.72	3.49	69.72 (57.01)	50.16
T3	Validamycin (0.1%)	33.94	3.23	64.55 (53.56)	53.93
T4	Tebuconazole (0.05%)	32.22	3.14	62.79 (52.62)	56.61
T5	Trifloxystrobin + Tebuconazole (0.05%)	37.20	2.97	59.36 (50.38)	48.66
T6	Azoxystrobin (0.05%)	31.24	2.81	56.20 (48.61)	58.35
T7	Pencycuron (0.1%)	44.83	3.91	77.73 (62.01)	47.12
T8	Control (water)	55.38	5.12	95.10 (77.20)	44.93
C. D. (5%)		12.42	1.19	14.60	N/A
C. V.		17.71	19.19	14.43	28.97

* Data of the table are the means of three replications, PDI: percent disease index. ^#^ Data within parentheses are angular transformed values. ^π^ Yield is calculated based on 3 m^2^ area of the experimental plots.

## Data Availability

Data is contained within the article and Supplementary Material.

## Supplementary Materials

The following supporting information can be downloaded at: https://www.mdpi.com/article/10.3390/cimb46040192/s1, Figure S1: trans-cinnamic acid standard curve; Figure S2: Lowry method standard curve; Figure S3: Standard curve of dextrose; Figure S4: Agrose gel picture of validated primes, Melt curve: Figures S5–S9 and Tables S1–S5. HSD Post Hoc analysis: Table S6.
