# Supplementary material for: Biochemical and Molecular Basis of Chemically Induced Defense Activation in Maize against Banded Leaf and Sheath Blight Disease
Source: Curr Issues Mol Biol. 2024 Apr 2;46(4):3063–80. doi: 10.3390/cimb46040192 (PMC11048768; doi:10.3390/cimb46040192)
Supplement: Supplementary file 1 [file cimb-46-00192-s001.zip › cimb-2854927-supplementary.pdf]

Supplemental : Annexure 1

Supplemental Figure S1:

Standard curve of trans-cinnamic acid (CA)

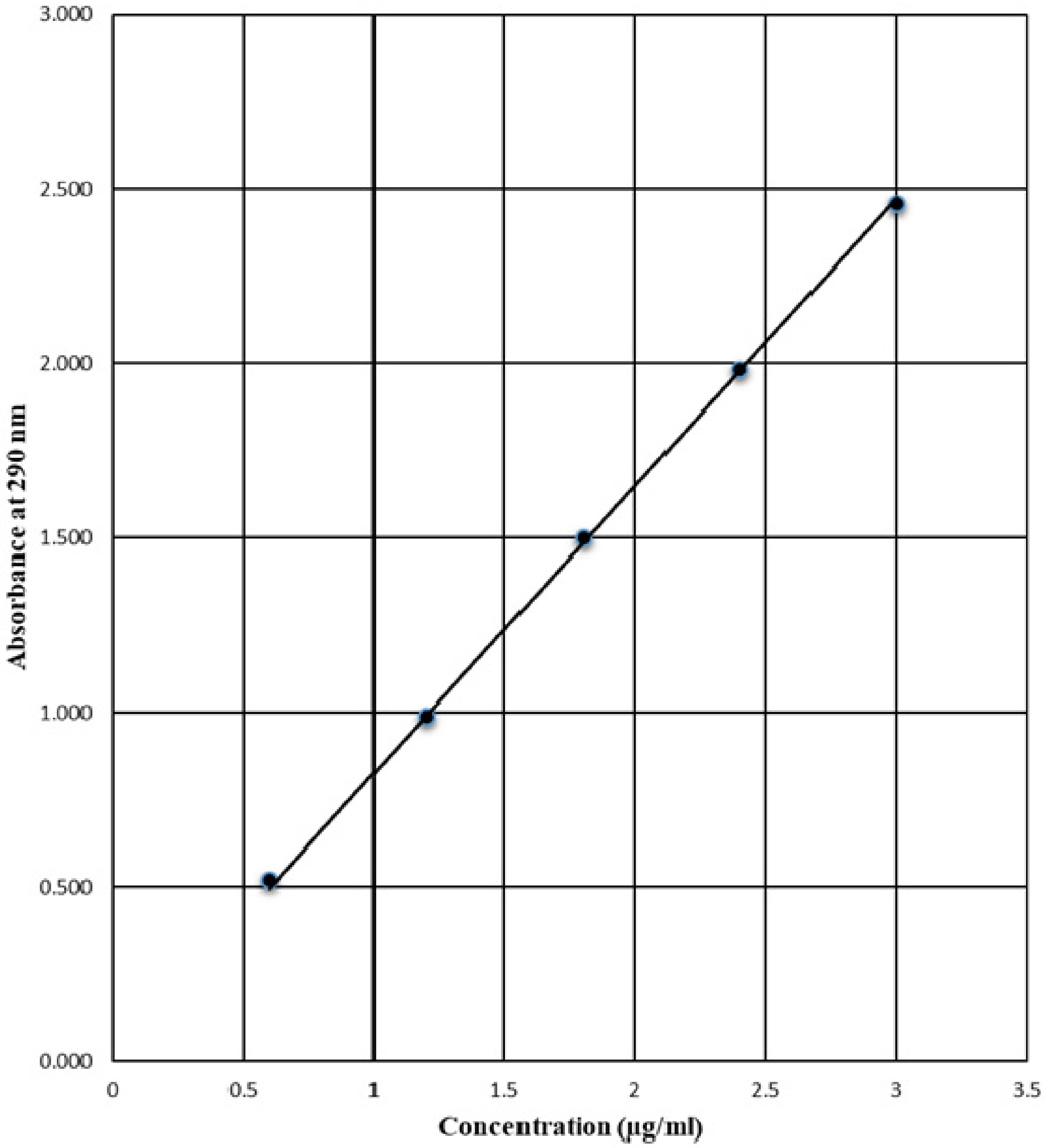

Figure S2: Lowry method Standard curve (bovine serum)

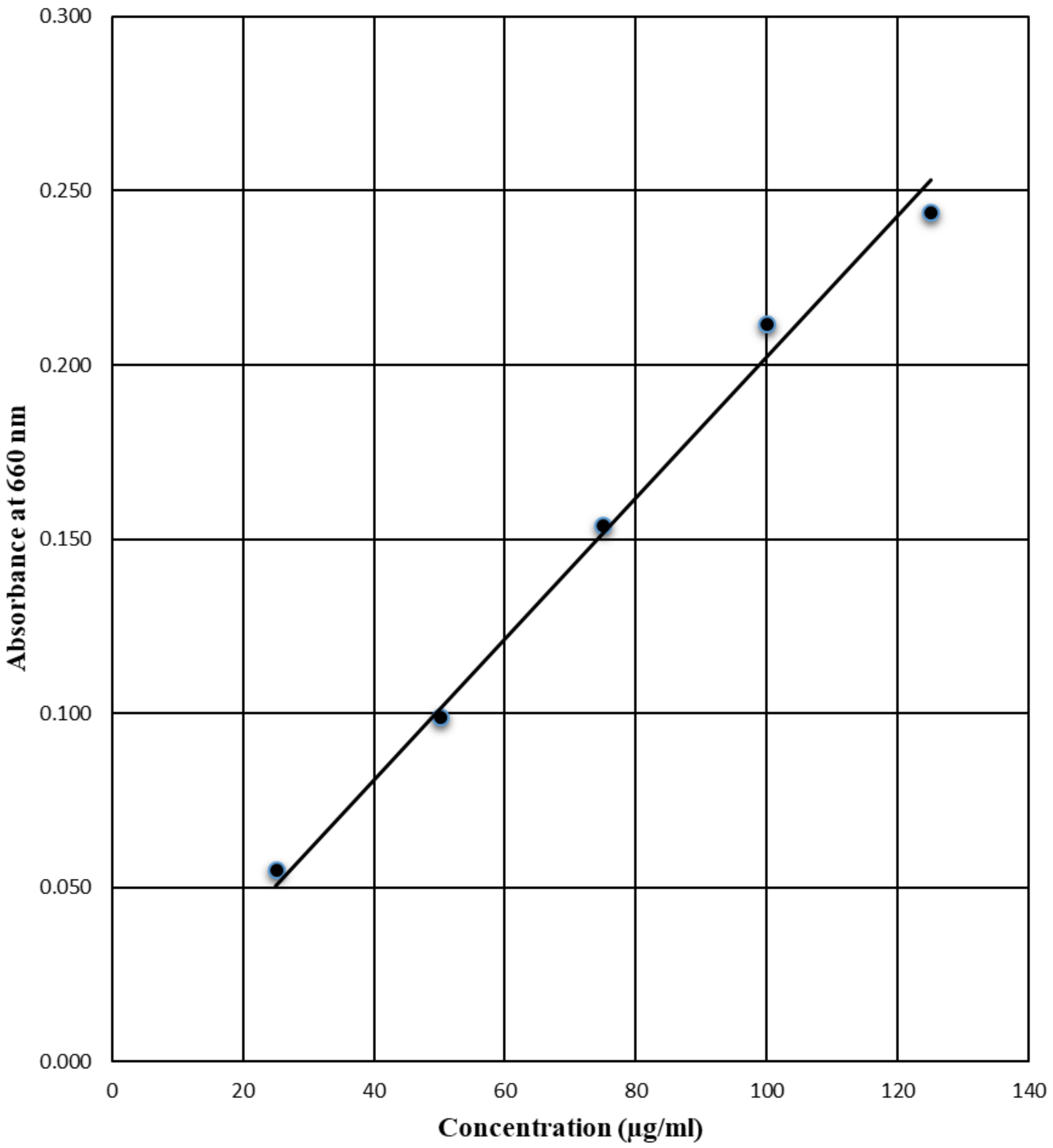

Supplemental Figure S3

Standard curve of dextrose (D-glucose)

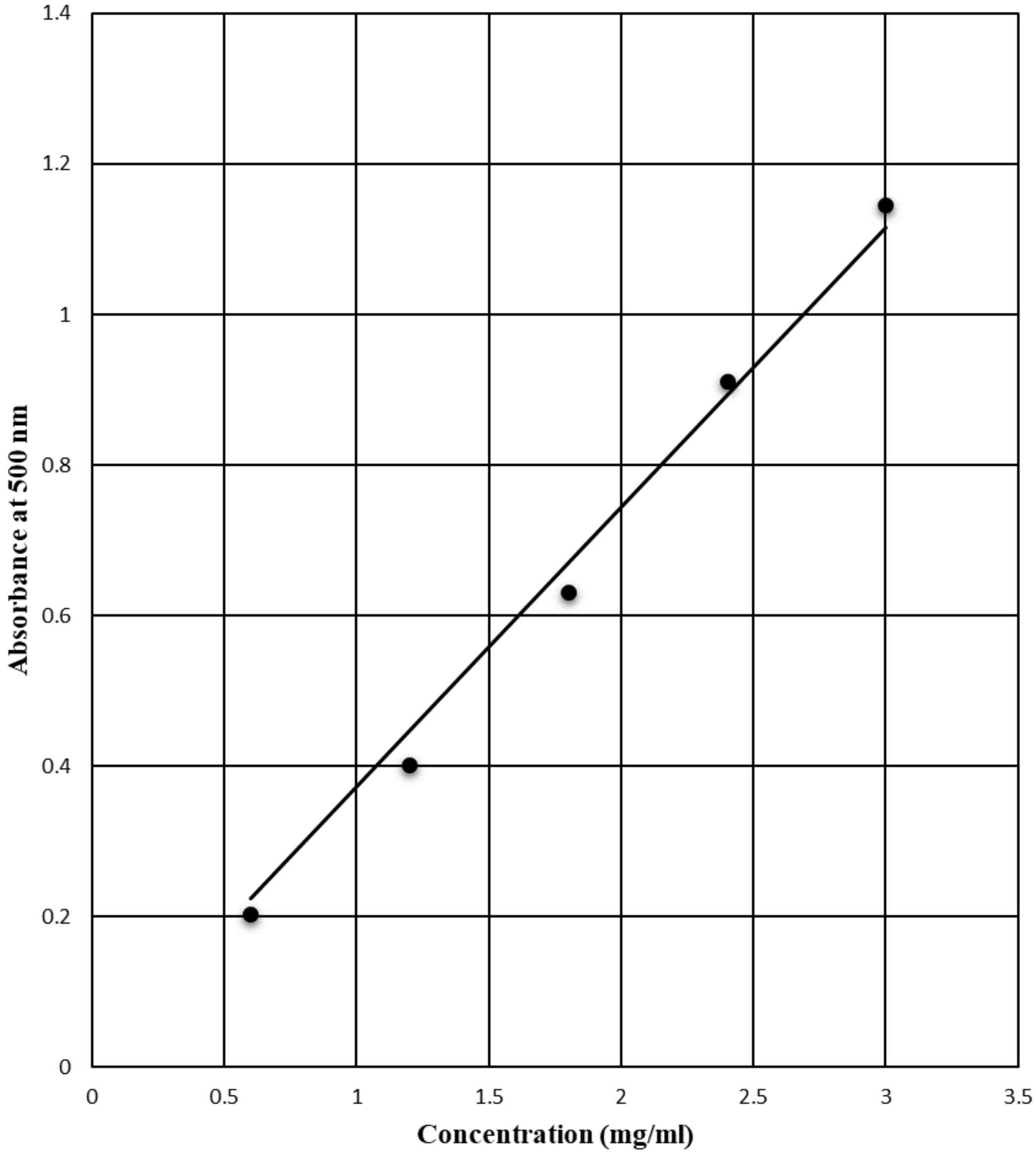

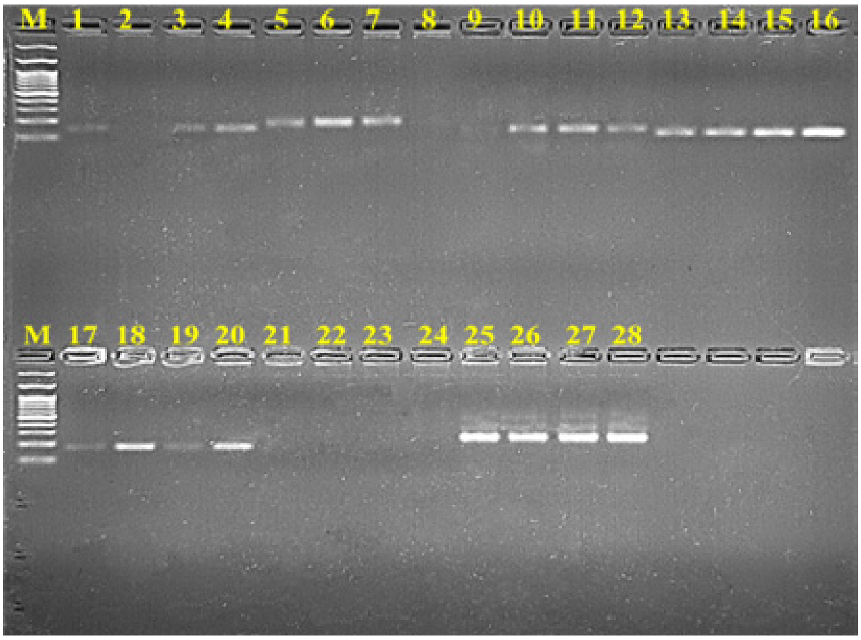

Fig. S4 Agarose gel showing amplicon of SOD (146bp, lane 1-4), *β*-1, 3-glucanase (188bp, lane 5-8), PPO (146bp, lane 9-12), APX (118pb, lane 13-16), CAT (180bp, lane 17-20), PAL (108bp, lane 21-24) and Actin (229bp, lane 25-28). M - 100bp ladder

Supplemental Figure S5

SOD gene melting curve (Actin + SOD)

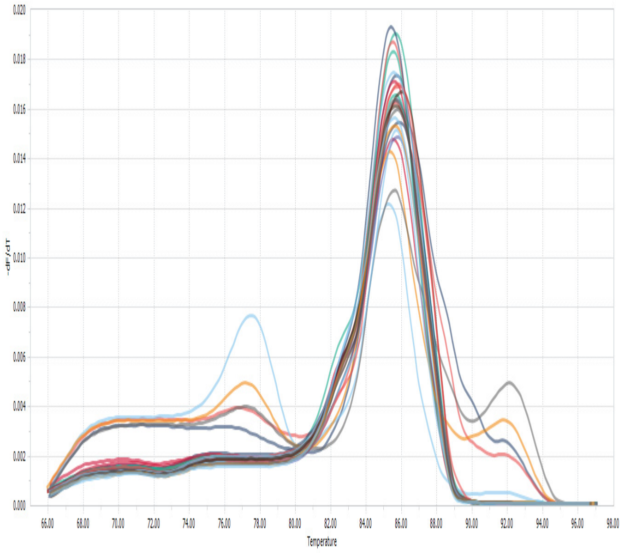

Table S1 Relative expression of maize SOD treated with plant defense inducers (*=average of three replicate)

Tr.	Treatments	Ct value* (Actin)	Ct value* (SOD)	Fold
T1	SA (50 ppm)	24.42	25.72	0.16
T2	SA (100 ppm)	23.20	29.10	0.01
T3	JA (50 ppm)	24.72	28.87	0.02
T4	JA (100 ppm)	25.25	25.97	0.24
T5	Check (water)	22.61	21.27	1.00
	**C. D. (5 %)**	**0.49**	**1.72**	
	**C. V.**	**1.11**	**3.56**	

Supplemental Figure S6

PPO gene melting curve (Actin + PPO)

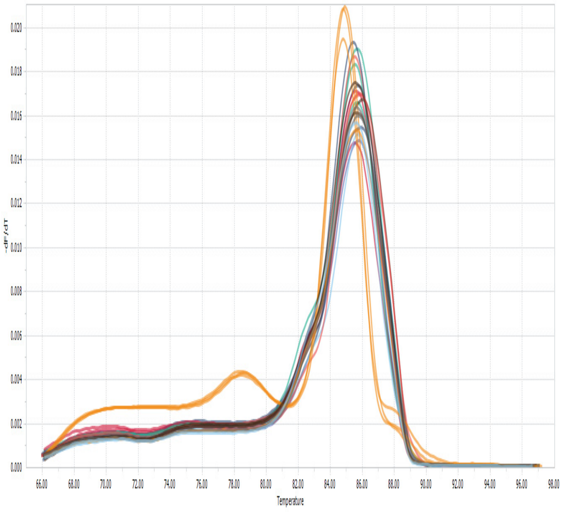

Table S2 Relative expression of maize PPO gene, treated with plant defense inducers (*=average of three replicate).

Tr.	Treatments	Ct value* (Actin)	Ct value* (PPO)	Fold
T1	SA (50 ppm)	24.42	31.47	0.80
T2	SA (100 ppm)	23.20	31.53	0.33
T3	JA (50 ppm)	24.72	31.39	1.04
T4	JA (100 ppm)	25.25	30.09	3.70
T5	Check (water)	22.61	29.34	1.00
	**C. D. (5 %)**	**0.49**	**0.25**	
	**C. V.**	**1.11**	**1.40**	

Supplemental Figure S7

APX gene melting curve (Actin + APX)

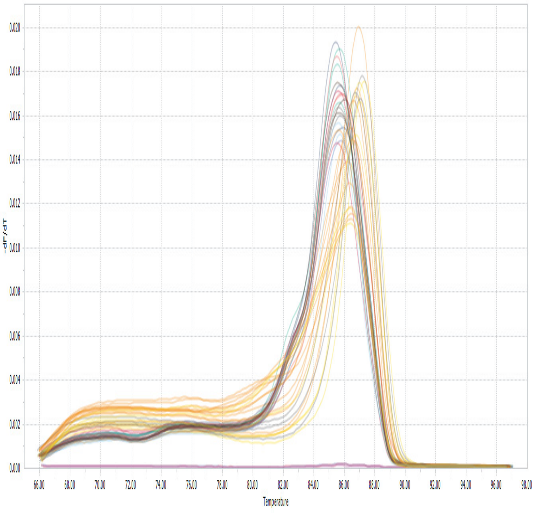

Table S3 Relative expression of maize APX gene, treated with plant defense inducers (*=average of three replicate).

Tr.	Treatments	Ct value* (Actin)	Ct value* (APX)	Fold
T1	SA (50 ppm)	24.42	27.36	0.75
T2	SA(100 ppm)	23.20	26.89	0.45
T3	JA (50 ppm)	24.72	26.29	1.94
T4	JA(100 ppm)	25.25	27.80	0.99
T5	Check(water)	22.61	25.14	1.00
	**C. D. (5 %)**	**0.49**	**1.51**	
	**C. V.**	**1.11**	**3.07**	

Supplemental Figure S8

CAT gene melting curve (Actin + CAT)

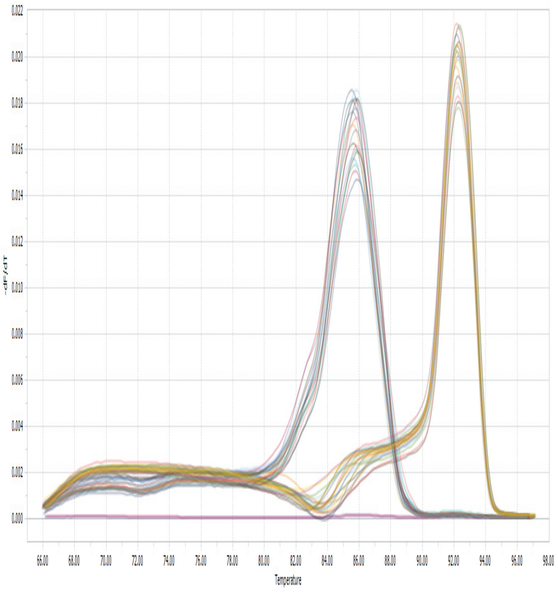

Table S4 Relative expression of maize CAT gene, treated with plant defense inducers (*=average of three replicate).

Tr.	Treatments	Ct value* (Actin)	Ct value* (CAT)	Fold
T1	SA (50 ppm)	25.18	27.22	3.06
T2	SA(100 ppm)	23.47	26.60	1.43
T3	JA (50 ppm)	25.33	27.30	3.21
T4	JA (100 ppm)	25.58	28.08	2.22
T5	Check (water)	22.52	26.16	1.00
	**C. D. (5 %)**	**0.67**	**0.62**	
	**C. V.**	**1.48**	**1.25**	

Supplemental Figure S9

B-1,3-glucanise gene melting curve
(Actin + B-1,3-gluc)

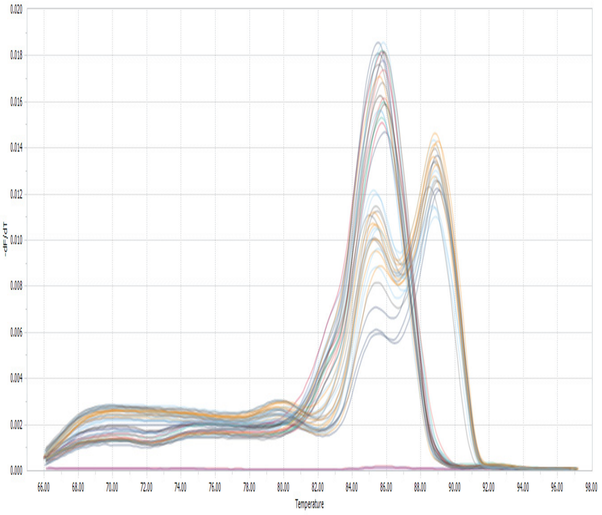

Table S5 Relative expression of maize PPO gene, treated with plant defense inducers (*=average of three replicate).

Tr.	Treatments	**Ct value*** (Actin)	**Ct value*** (β-1, 3-gluc)	**Fold**
T1	SA(50 ppm)	25.18	20.94	6.73
T2	SA(100ppm)	23.47	24.10	0.23
T3	JA (50 ppm)	25.33	21.91	3.82
T4	JA(100 ppm)	25.58	19.32	27.35
T5	Check(water)	22.52	21.03	1.00
	**C. D. (5 %)**	**0.67**	**0.96**	
	**C. V.**	**1.48**	**2.42**	
