# Supplementary material for: Biochemical and Molecular Basis of Chemically Induced Defense Activation in Maize against Banded Leaf and Sheath Blight Disease
Source: Curr Issues Mol Biol. 2024 Apr 2;46(4):3063–80. doi: 10.3390/cimb46040192 (PMC11048768; doi:10.3390/cimb46040192)
Supplement: Supplementary file 1 [file cimb-46-00192-s001.zip › cimb-2854927-Table S6.pdf]

Table S6: Tukey HSD / Tukey Kramer

ANOVA 5% level of significance and Post-hoc Tukey HSD Test statistics reveal that treatment groups significantly differ from groups. This comparative analysis offers insights into susceptibility and resistance levels across the treatments (F Statistic =10.1453, P-value 0.0243).

Source	DF	Sum of Square	Mean Square	F Statistic	P-value
**Groups** (between groups)	3	834609.375	278203.125	10.1453	0.0243
**Error** (within groups)	4	109687.5004	27421.8751		
**Total**	7	944296.8754	134899.5536		

Pair	Difference	SE	Q	Lower CI	Upper CI	Critical Mean	p-value
x1-x2	262.5	117.0937	2.2418	-411.6153	936.6153	674.1153	0.4755
x1-x3	562.5	117.0937	4.8038	-111.6153	1236.6153	674.1153	0.08738
x1-x4	862.5	117.0937	7.3659	188.3847	1536.6153	674.1153	0.02189
x2-x3	300	117.0937	2.5621	-374.1153	974.1153	674.1153	0.3854
x2-x4	600	117.0937	5.1241	-74.1153	1274.1153	674.1153	0.072
x3-x4	300	117.0937	2.5621	-374.1153	974.1153	674.1153	0.3854

Group	x2	x3	x4
x1	262.5	562.5	862.5
x2	0	300	600
x3	300	0	300
